# Analysis of six chloroplast genomes provides insight into the evolution of *Chrysosplenium* (Saxifragaceae)

**DOI:** 10.1186/s12864-020-07045-4

**Published:** 2020-09-10

**Authors:** Zhihua Wu, Rui Liao, Tiange Yang, Xiang Dong, Deqing Lan, Rui Qin, Hong Liu

**Affiliations:** 1 Hubei Provincial Key Laboratory for Protection and Application of Special Plant Germplasm in Wuling Area of China, Key Laboratory of State Ethnic Affairs Commission for Biological Technology, College of Life Sciences, South-Central University for Nationalities, Wuhan, 430074 Hubei China; 2 CAS Key Laboratory of Plant Germplasm Enhancement and Specialty Agriculture, Wuhan Botanical Garden, Chinese Academy of Sciences, Wuhan, 430074 Hubei China

**Keywords:** Saxifragaceae, *Chrysosplenium*, Chloroplast genome, Opposite leaves, Alternate leaves, Phylogenomics

## Abstract

**Background:**

*Chrysosplenium* L. (Saxifragaceae) is a genus of plants widely distributed in Northern Hemisphere and usually found in moist, shaded valleys and mountain slopes. This genus is ideal for studying plant adaptation to low light conditions. Although some progress has been made in the systematics and biogeography of *Chrysosplenium*, its chloroplast genome evolution remains to be investigated.

**Results:**

To fill this gap, we sequenced the chloroplast genomes of six *Chrysosplenium* species and analyzed their genome structure, GC content, and nucleotide diversity. Moreover, we performed a phylogenetic analysis and calculated non-synonymous (Ka) /synonymous (Ks) substitution ratios using the combined protein-coding genes of 29 species within Saxifragales and two additional species as outgroups, as well as a pair-wise estimation for each gene within *Chrysosplenium*. Compared with the outgroups in Saxifragaceae, the six *Chrysosplenium* chloroplast genomes had lower GC contents; they also had conserved boundary regions and gene contents, as only the *rpl32* gene was lost in four of the *Chrysosplenium* chloroplast genomes. Phylogenetic analyses suggested that the *Chrysosplenium* separated to two major clades (the opposite group and the alternate group). The selection pressure estimation (Ka/Ks ratios) of genes in the *Chrysosplenium* species showed that *matK* and *ycf2* were subjected to positive selection.

**Conclusion:**

This study provides genetic resources for exploring the phylogeny of *Chrysosplenium* and sheds light on plant adaptation to low light conditions. The lower average GC content and the lacking gene of *rpl32* indicated selective pressure in their unique habitats. Different from results previously reported, our selective pressure estimation suggested that the genes related to photosynthesis (such as *ycf2*) were under positive selection at sites in the coding region.

## Background

Challenging environments may impose selective pressure on genes, which could leave a footprint of natural selection in genes involved in adaptation to the environment. The chloroplast genome is typically quadripartite in structure, containing a large single copy (LSC) and a small single copy (SSC) separated by a pair of inverted repeats (IR). It is widely used for chloroplast inheritance, domestication studies, phylogeny and adaptative evolution [1–3]. Adaptive evolution is considered as the improved adaptability of species for changing environmental conditions during their evolutionary processes. And it is driven by evolutionary processes such as natural selection, which act on genetic variations produced by genetic recombination, gene mutations, and gene flow [4]. Strong purifying selection detected rather than expected positive selection in chloroplast genome of the green alga (*Ostreobium quekettii*) facilitated its extremely low light adaptation [5]. This study contributed to our understanding of plant adaptive evolution. However, to our knowledge, a comparative analysis of chloroplast genomes of angiosperms with low light requirements has not been conducted.

*Chrysosplenium* L. is a genus of Saxifragaceae and belongs to the subfamily Saxifragoideae according to the APG IV [6]. The genus plays an important role in the phylogeny of Saxifragaceae, comprises about 79 perennial herbs [7], and mainly occurs in the northern hemisphere, with the highest species diversity in East Asia; only two species, *Chrysosplenium valdivicum* Hook and *Chrysosplenium macranthum* Hook, are found in the Southern Hemisphere [8, 9]. Thirty-six species and fifteen variants have been recorded in China [10, 11]. *Chrysosplenium* is divided into two subgenera according to the leaf arrangement: *Alternifolia* Franchet with alternate leaves and *Oppositifolia* Franchet with opposite leaves [8, 10, 12]. The genus was regarded as a typical group with floristic disjunction and is important for studying speciation [13, 14]. *Chrysosplenium* (Saxifragaceae) is usually found in shaded valleys and mountain slopes and, compared with other genera within Saxifragaceae, this genus has the lowest light requirement [8, 15]. Therefore, examination of the chloroplast genomes of *Chrysosplenium* species may provide insight into the impacts of low light in angiosperms.

Nuclear markers (such as the internal transcribed spacer, ITS) of the ribosomal DNA, and chloroplast markers were employed to determine the molecular phylogeny of *Chrysosplenium* [16, 17]*.* Compared to nuclear markers, the chloroplast genome possesses highly conserved DNA sequences and a lower substitution level (especially in inverted repeat regions). Therefore, the chloroplast genome is ideal for phylogenetic inference at the species and higher levels [9, 17–20].

In this study, we aimed to provide a comprehensive insight into the evolution of the chloroplast genomes of several *Chrysosplenium* species. First, we sequenced the chloroplast genomes of six *Chrysosplenium* species in addition to a previous study [21]. Next, we conducted comparative chloroplast genome analyses for these six genomes, plus four Saxifragaceae chloroplast genomes from GenBank. Then, we constructed a phylogeny of *Chrysosplenium* using chloroplast genomes of 29 species within Saxifragales and two outgroups. Finally, we estimated selective pressures to investigate whether the genes related to photosynthesis in *Chrysosplenium* are under purifying selection or positive selection.

## Results

### Organization of the Chloroplast Genomes of *Chrysosplenium* species

**Fig. 1:**
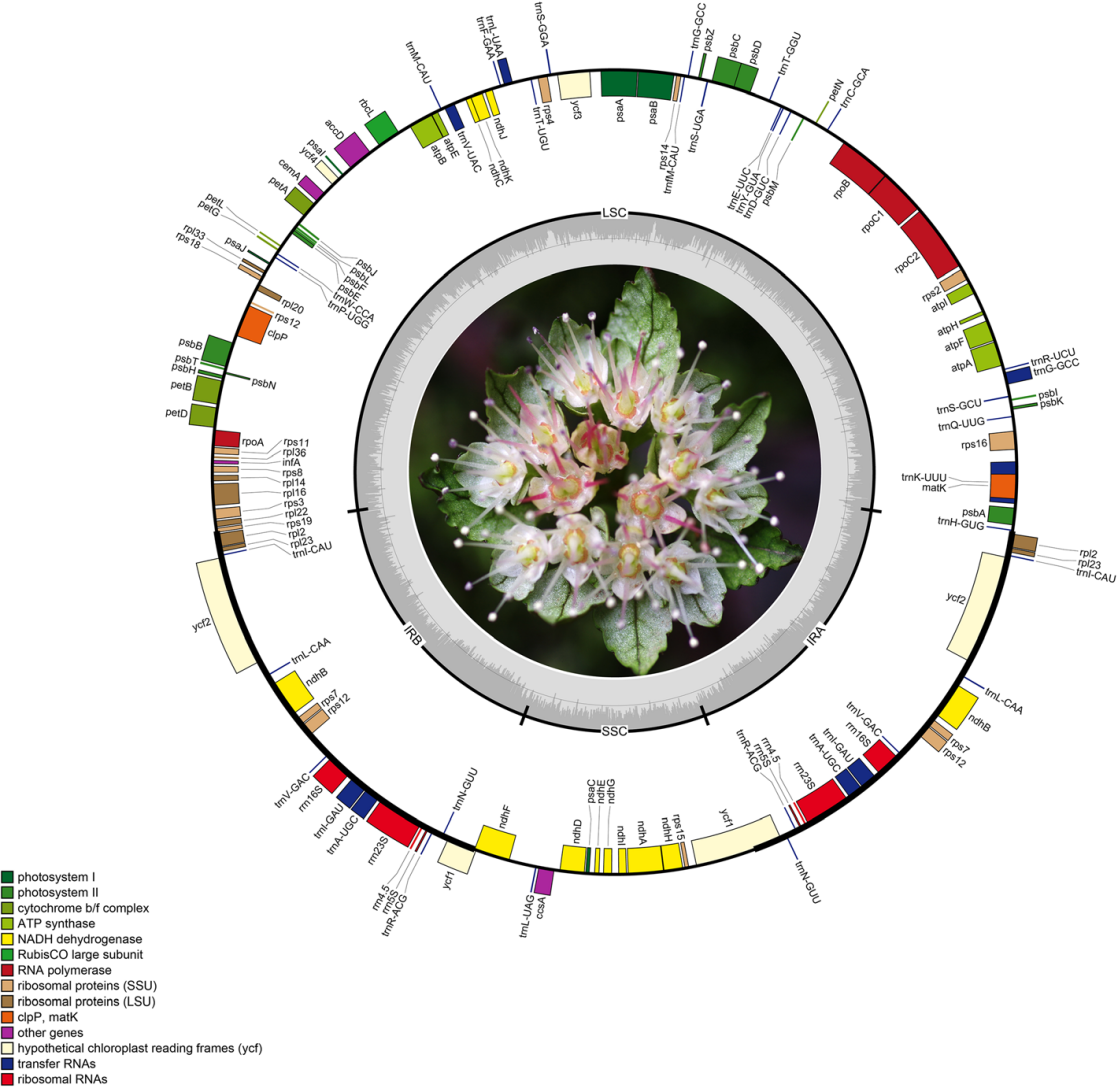
Gene map of the *Chrysosplenium macrophyllum* chloroplast genomes. Genes inside the circle are transcribed clockwise, genes outside are transcribed counter-clockwise. Genes are color-coded to indicate functional groups. The dark gray area in the inner circle corresponds to GC content while the light gray corresponds to the adenine-thymine (AT) content of the genome. The small (SSC) and large (LSC) single copy regions and inverted repeat (IRa and IRb) regions are noted in the inner circle

The chloroplast genomes of the *Chrysosplenium* species contain the typical quadripartite structures (Fig. 1), which include a large single copy (LSC), two inverted repeats (IR) and a small single copy (SSC) region. The seven *Chrysosplenium* chloroplast genomes ranged from 151,679 bp to 153,460 bp in length (see Table 1 for details), with IRs 25,974–26,224 bps, LSCs 82,771–83,752 bps and SSCs 16,960–17,342 bps. Each *Chrysosplenium* chloroplast genome encoded 30 transfer RNAs (tRNAs) and 4 ribosomal RNAs (rRNAs). Each genome also includes 79 functional proteins (Table 2) encoding genes except for *C. macrophyllum*, *C. flagelliferum*, *C. alternifolium*, *C. ramosum*, which lacked *rpl32*. By homolog and expression analysis of chloroplast *rpl32* in *C. sinicum* (*Cp_rpl32*), another homolog (*Nu_rpl32*) was also identified nuclear genome of *C. sinicum*, and the expression value (4.86 FPKMs) of *Nu_rpl32* is much lower than that (20,835.9 FPKMs) of *Cp_rpl32* (Additional file 1: Supplementary Figure S1)*.* In total, each chloroplast genome includes 113 (*rpl32* present) or 112 (*rpl32* loss) genes. The *rps12* gene in *Chrysosplenium* was recognized as a trans-spliced gene, with the first exon located in the LSC region and the other one or two exons distributed in the IR regions. In addition, 17 intron-containing genes were also detected (Additional file 2: Supplementary Table S4). The chloroplast genome size and gene content neither significantly diverged between *Oppositifolia* and *Alternifolia* subgenera (Table 1) nor significantly diverged between *Chrysosplenium* and other genera of Saxifragaceae.

**Table 1:** General information and comparison of chloroplast genomes of Saxifragaceae species

Characteristic	*C. macrophyllum*	*C. flagelliferum*	*C. alternifolium*	*C. kamtschaticum*	*C. ramosum*	*C. sinicum*	*C. aureobracteatum*
Size (base pair, bp)	152,837	151,679	152,619	152,561	153,460	153,427	153,102
LSC length (bp)	83,583	82,771	83,524	83,175	83,670	83,745	83,753
SSC length (bp)	17,264	16,960	17,111	16,986	17,342	17,236	17,317
IR length (bp)	25,995	25,974	25,992	26,200	26,224	26,223	26,016
Number of genes	112	112	112	113	112	113	113
Protein-coding genes	78	78	78	79	78	79	79
rRNA genes	4	4	4	4	4	4	4
tRNA genes	30	30	30	30	30	30	30
LSC GC%	35.33	35.26	35.35	35.28	35.24	35.05	35.20
SSC GC%	31.42	31.37	31.40	31.46	31.64	31.27	31.16
IR GC%	42.89	42.87	42.86	42.71	42.69	42.75	42.85
Lacking gene	*rpl32*	*rpl32*	*rpl32*		*rpl32*

**Table 2:** Genes encoded in the *C. macrophyllum* chloroplast genome

Group of Genes	Gene Name
tRNA genes	trnH-GUG trnK-UUU* trnQ-UUG trnS-GCU trnG-GCC* trnR-UCU trnC-GCA trnD-GUC trnY-GUA trnE-UUC trnT-GGU trnS-UGA trnG-GCC trnfM-CAU trnS-GGA trnT-UGU trnL-UAA* trnF-GAA trnV-UAC* trnM-CAU trnW-CCA trnP-UGG trnI-CAU(× 2) *trnL-CAA*(× 2) *trnV-GAC*(× 2) trnI-GAU*(× 2) trnA-UGC*(× 2) *trnR-ACG*(× 2) *trnN-GUU*(× 2) *trnL-UAG*
rRNA genes	*rrn16(× 2) rrn23(× 2) rrn4.5(× 2) rrn5(× 2)*
Ribosomal small subunit	rps16* rps2 rps14 rps4 rps18 rps12**(× 2) rps11 rps8 rps3 rps19 rps7(× 2) rps15
Ribosomal large subunit	rpl33 rpl20 rpl36 rpl14 rpl16* rpl22 rpl2(× 2) rpl23(× 2)
DNA-dependent RNA polymerase	rpoC2 rpoC1* rpoB rpoA
Photosystem I	*psaB psaA psaI psaJ psaC*
Large subunit of rubisco	*rbcL*
Photosystem II	*psbA psbK psbI psbM psbC psbZ psbG psbJ psbL psbF psbE psbB psbT psbN psbH*
NADH dehydrogenase	ndhJ ndhK ndhC ndhB*(×2) ndhF ndhD ndhE ndhG ndhI ndhA* ndhH
Cytochrome b/f complex	petN petA petL petG petB* petD*
ATP synthase	atpA atpF* atpH atpI atpE atpB
Maturase	*matK*
Subunit of acetyl-CoA carboxylase	*accD*
Envelope membrane protein	*cemA*
Protease	clpP**
Translational initiation factor	*infA*
C-type cytochrome synthesis	*ccsA*
Conserved open reading frames (ycf)	ycf3** ycf4 ycf2(×2) ycf1(× 2)

Genes with one or two introns are indicated by one (*) or two asterisks (**), respectively. Genes in the IR regions are followed by the (× 2) symbol.

### GC content, nucleotide diversity, and repeat analysis

**Fig. 2:**
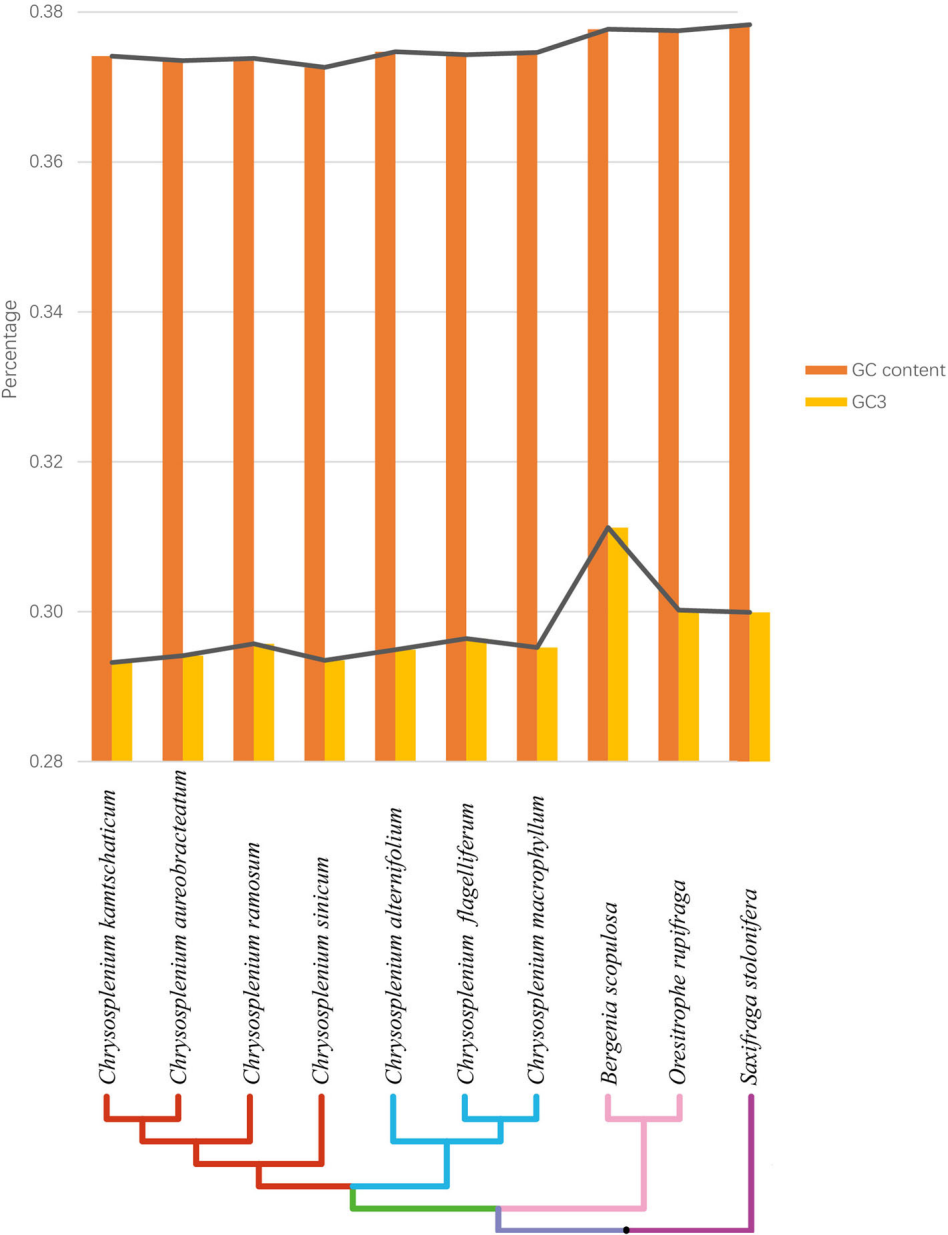
Changes in plastid GC content of Saxifragaceae. This graph shows the total GC content (orange bar and black line) and the third codon position GC content (yellow bar and gray line) of each species. The subgenera of *Oppositifolia* and *Alternifolia* are colored by red and blue, respectively

When we compared the total GC content of the chloroplast genomes of *Chrysosplenium* species with that of the chloroplast genomes of the three non-*Chrysosplenium* Saxifragaceae species (*S. stolonifera*, *B. scopulosa*, and *O. rupifraga*), we found the *Chrysosplenium* species have the lowest total GC contents (< 37.5%) (Fig. 2 and Additional file 2: Supplementary Table S5). In addition, *Chrysosplenium* has the lowest GC contents (< 29.7%) at the third codon position (GC3). Within the *Chrysosplenium* species, the GC contents in subgenus *Oppositifolia* were slightly lower than those in subgenus *Alternifolia*, regardless of the total GC contents or those in GC3.

The IR regions were more conserved than the LSC and SSC regions, with average Pi values of 0.00586 in IR regions, 0.01760 in the LSC region, and 0.01900 in the SSC region (Additional file 2: Supplementary Table S6 and Additional file 3: Supplementary Figure S2). In the LSC region, *psbT* has the highest Pi value of 0.22159, followed by *trnG*-GCC with Pi value of 0.10369.

Among the mono-, di-, tri-, tetra-, penta-, and hexa-nucleotide categories of SSRs in the chloroplast genomes of the *Chrysosplenium* species, mono-nucleotide repeats were the most common (Additional File 4: Supplementary Table S7 and Additional File 5: Supplementary Figure S3A) ranging from 42.42% (*C. sinicum*) to 61.29% (*C. flagelliferum*). Hexa-nucleotide repeats account for the lowest proportion of SSRs in *C. ramosum*, *C. sinicum*, and *C. flagelliferum*. *Chrysosplenium* species contained fewer SSRs than *B. scopulosa* and *O. rupifraga*. Among the four repeat types, the most common repeat type was palindromic repeats, which ranged from 53.13% in *C. aureobracteatum* to 42.42% in *C. macrophyllum* (Additional File 4: Supplementary Table S8 and Additional File 5: Supplementary Figure S3B).

### Boundary regions and comparative analysis

**Fig. 3:**
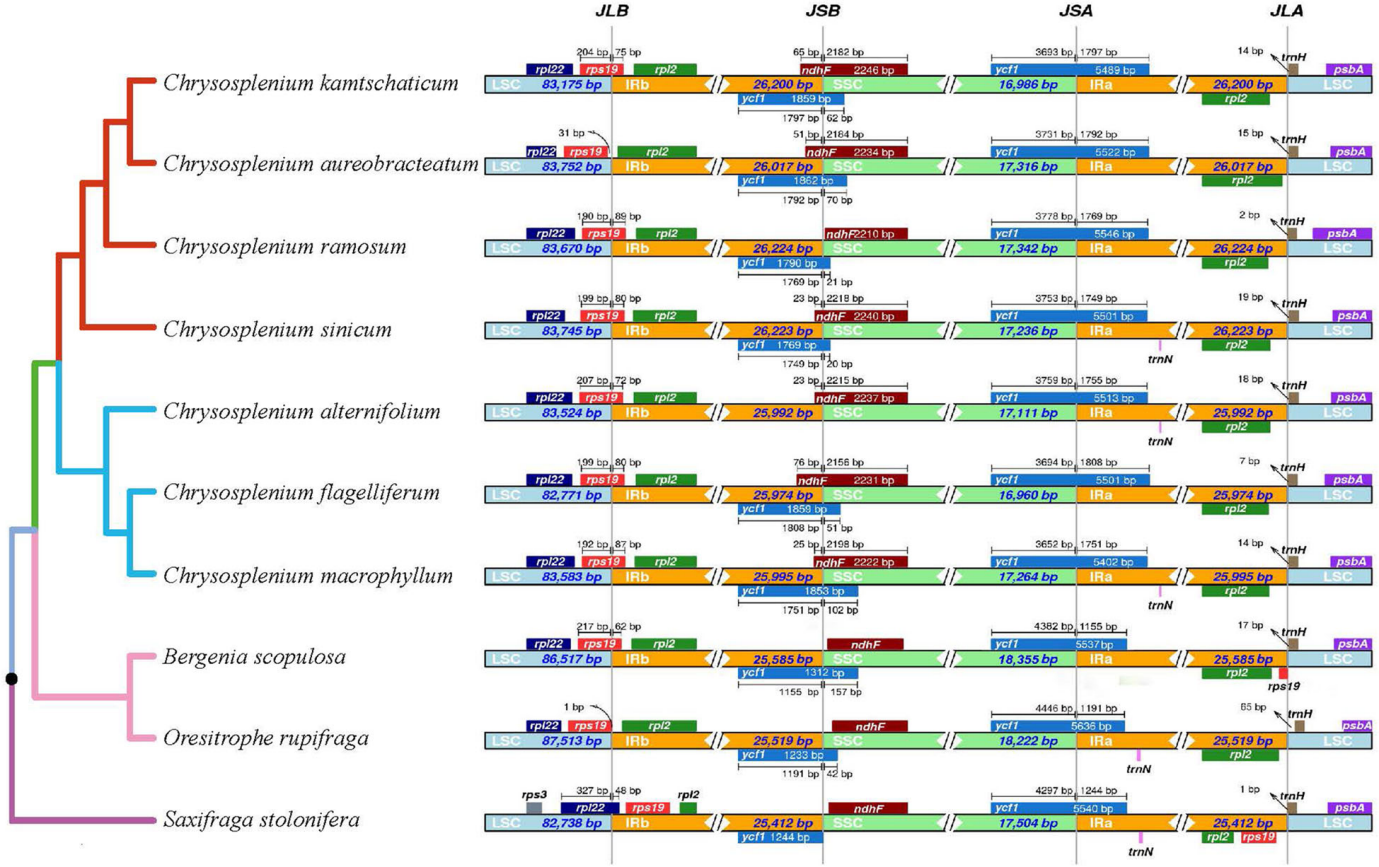
Comparison of the borders of the LSC, SSC, and IR regions among ten chloroplast genomes. JLB, JSB, JSA and JLA denote the junction sites of LSC/IRb, IRb/SSC, SSC/IRa and IRa/LSC, respectively

When comparing the chloroplast genomes of *Chrysosplenium* species, we found that IR/LSC junctions of IRb are largely located between *rpl2* and *rps19* (Fig. 3). Moreover, the overlap of *ycf1* pseudogenes and *ndhF* appeared in different locations among the *Chrysosplenium* species: in the region of the SSC for *C. ramosum*, and at the IRb/SSC border for the other five species. *C. alternifolium* did not contain the *ycf1* pseudogene. The *ycf1* genes were sited at the SSC/IRa boundary and the length of *ycf1* ranged from 5402 to 5546 bps. The *trnH* genes of the seven *Chrysosplenium* species were located in the LSC region, 2–19 bp away from the IRa–LSC border.

When comparing the genome boundaries of the *Chrysosplenium* species to the other three non-*Chrysosplenium* species of Saxifragaceae, *ndhF* was at the IRb/SSC boundary in most species of *Chrysosplenium*, except for *C. ramosum*, which showed contraction of the SSC and expansion of IRb. In addition, *S. stolonifera* was slightly different from the other two non-*Chrysosplenium* species in Saxifragaceae. In *S. stolonifera*, the contraction of the LSC region resulted in the *rpl22* gene being at the IRb/LSC junction, which placed the whole *rps19* gene in the IRb region. The *rps19* pseudogenes were also found in the IRa region in *S. stolonifera* and *B. scopulosa*. When these data were combined with the phylogenetic tree of the three clades (*S. stolonifera*, *B. scopulosa* and *O. rupifraga*, and *Chrysosplenium*) inferred from whole-chloroplast protein-coding genes (Fig. 3), we found that the chloroplast genome structure within *Chrysosplenium* species is not strongly conserved, although the gene content is conserved.

**Fig. 4:**
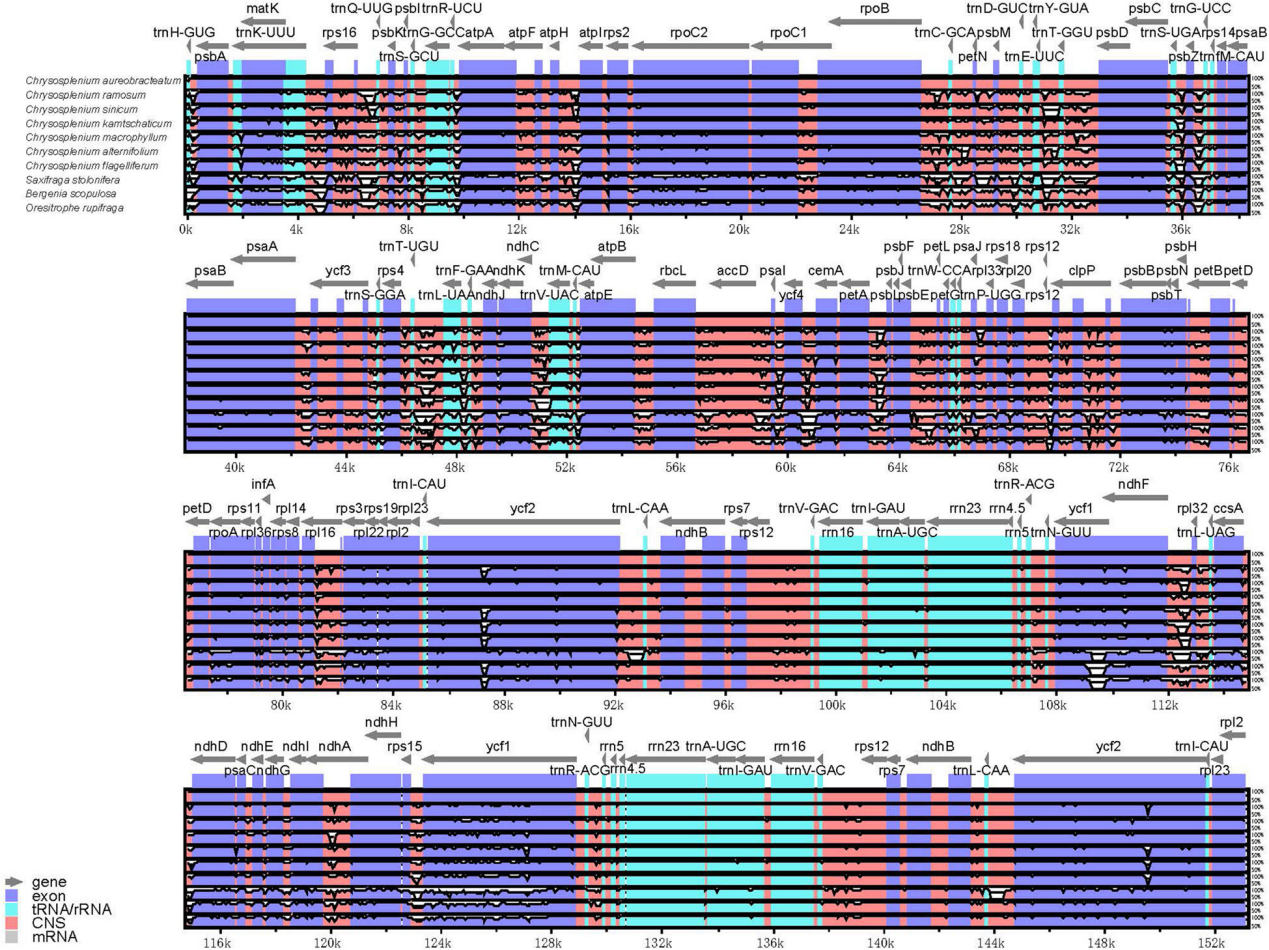
The comparative analysis with LAGAN program of the whole-chloroplast genome of seven different species from the family of Saxifragaceae. The percentage of identity is shown in the vertical axis, ranging from 50 to 100%, while the horizontal axis shows the position within the chloroplast genome. Each arrow displays the annotated genes and direction of their transcription in the reference genome (*C. aureobracteatum*). Genome regions are color-coded as exon, tRNA, conserved noncoding sequences (CNS), and mRNA

LAGAN and Shuffle-LAGAN gave very similar results in the genetic divergence among the chloroplast genomes of Saxifragaceae species (Fig. 4 and Additional File 6: Supplementary Figure S4). The chloroplast genomes of the *Chrysosplenium* species were more conserved when compared with the three non-*Chrysosplenium* species of Saxifragaceae, and the intergenic spacer (IGS) regions had the highest levels of divergence: *trnK*–*rps16*, *rps16*–*trnQ*, *rpoB*–*trnC*, *petN*–*psbM*, *trnT*–*psbD*, *psbZ*–*trnG*, *trnT*–*trnL*, *accD*–*psaI*, *ycf4*–*cemA*, *ndhF*–*rpl32*, and *rps15*–*ycf1*. In addition, we found some highly variable coding sequences (*ndhD*, *ycf2*, *ndhA*, and *ycf1*), and the IR regions were more conserved than LSC and SSC regions in all the species tested. We also found slight difference for *rpoC2*, *ycf2*, and *ycf1*, which correspond to the difference between the *Alternifolia* and *Oppositifolia* subgenera.

### Selective pressure analyses

**Fig. 5:**
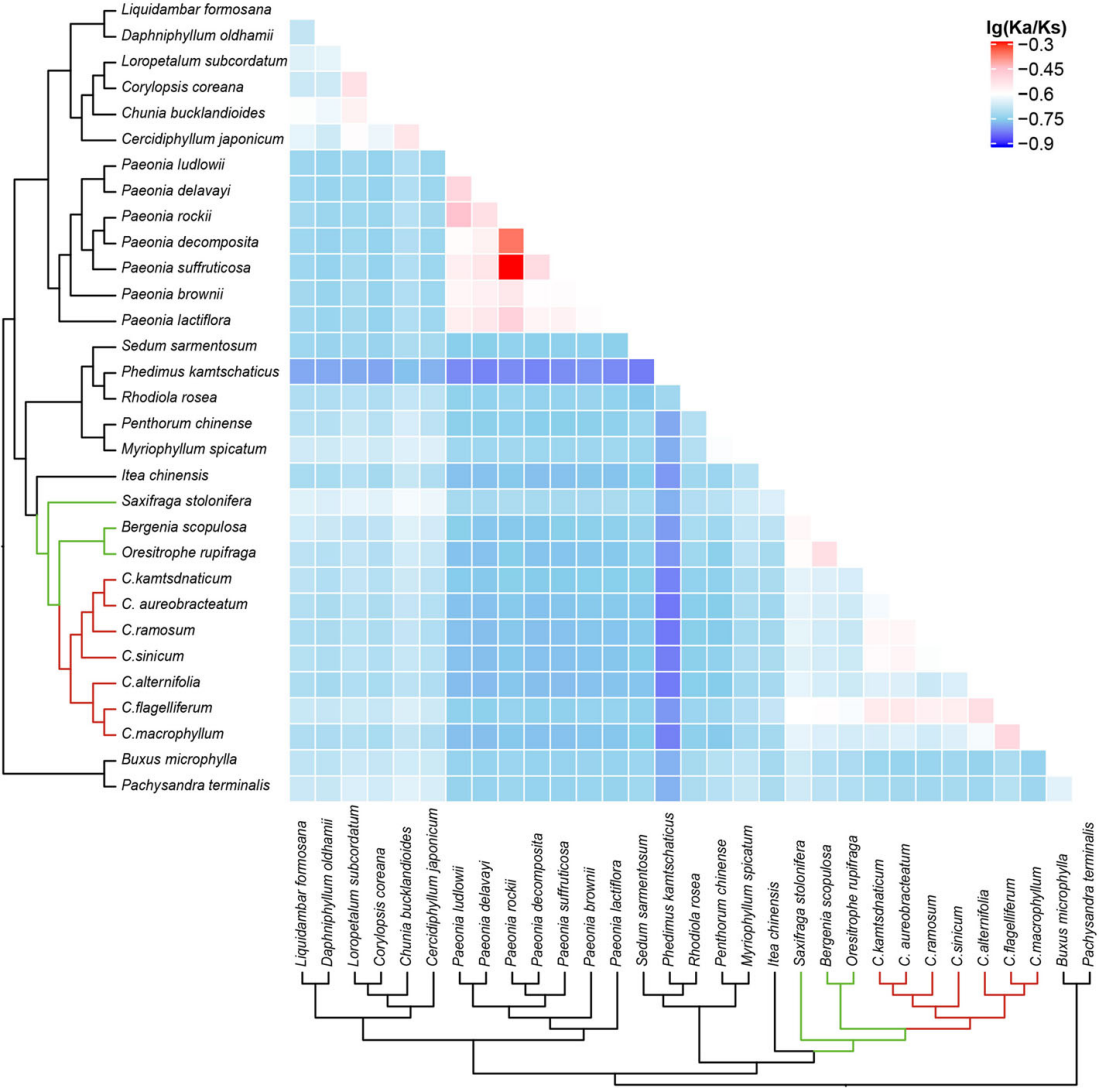
Pairwise Ka/Ks ratios in Saxifragaceae and other families. This heatmap shows pairwise Ka/Ks ratios between every sequence in the multigene nucleotide alignment. *Chrysosplenium* is shown on red branches. The scale factors associated with each value are shown on the top right side of the figure

We calculated the Ka/Ks ratios, the ratios of the rate of non-synonymous substitutions (Ka) to the rate of synonymous substitutions (Ks), at the species level by concatenating all of the 79 genes into a super-matrix. In *Chrysosplenium* species, the Ka/Ks ratios were around 0.2. This result suggested that at the whole-chloroplast protein level, the *Chrysosplenium* species have been subjected to a stronger purifying selection (Fig. 5, Additional File 4: Supplementary Table S9 and Additional File 4: Supplementary Table S10).

**Fig. 6:**
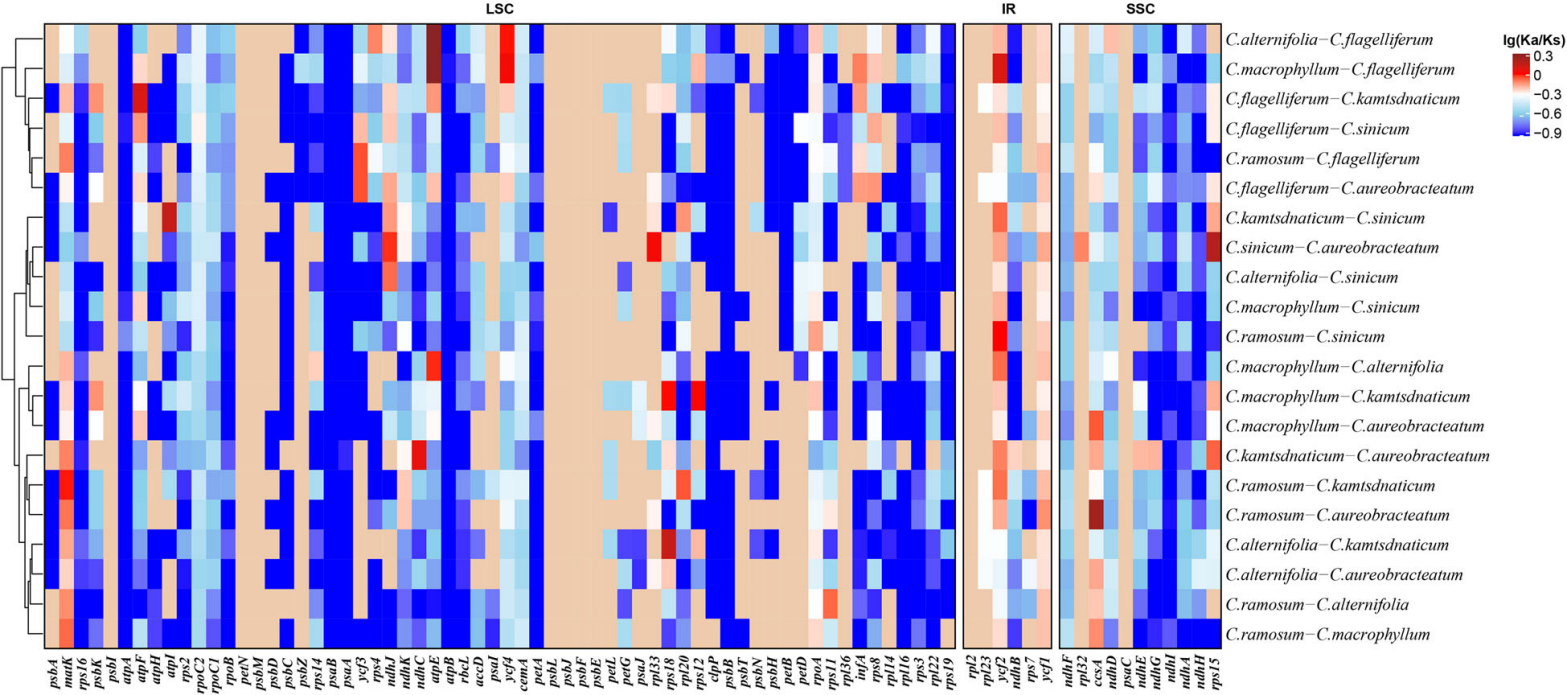
Pairwise Ka/Ks ratios in *Chrysosplenium* in different genes. This heatmap shows pairwise Ka/Ks ratios among each individual gene in the *Chrysosplenium* species. The scaled Ka/Ks ratios are shown on the top right. The color closer to red represents the gene has a high Ka/Ks ratio

The Ka/Ks ratios were also calculated for all of the 79 protein-coding genes of the ten chloroplast genomes of *Chrysosplenium* separately (Fig. 6 and Additional File 4: Supplementary Table S7). Two genes (*matK*, *ycf2*) had Ka/Ks ratios around 1.0 in most species, implying possible positive selection. Specially, *matK* showed an average Ka/Ks ratio of 0.74 when compared with *C. ramosum*. Among the *Chrysosplenium* species, *ycf2* often had a ratio higher than 0.8. Most of the other genes had a Ka/Ks ratio range from 0.1–0.3, implying strong purification (Additional File 4: Supplementary Table S10).

**Fig. 7:**
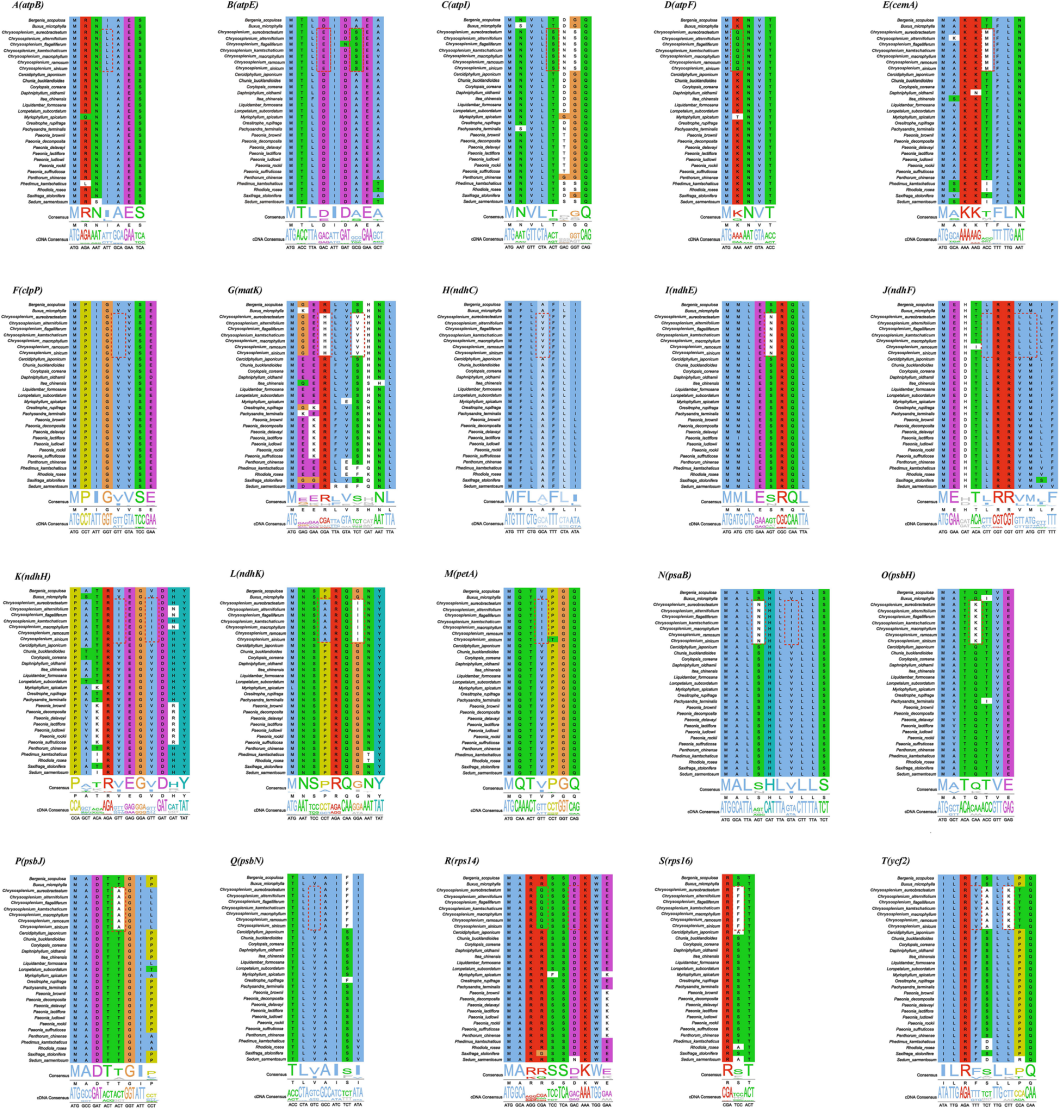
The partial alignment of 19 genes suggesting sites with positive selection in the BEB test. The red blocks stand for the amino acids in *Chrysosplenium* with a high BEB posterior probability

Sixty-six single-copy genes were used for selective pressure estimation with the branch-site model (Additional File 4: Supplementary Table S9 and Supplementary Table S11). We found that *matK* was positively selected in *Chrysosplenium* with the *p*-value = 0.022 and the Bayes Empirical Bayes (BEB) posterior probability for one amino acid site (117S, from polar Ser to non-polar Val) larger than 0.972. And the gene of *ycf2* was also positively selected in *Chrysosplenium* with *p*-value = 0.00003 and the BEB posterior probability for 0.953 in 1028 K (from Lys to Leu). In addition, positively selected sites were detected for 18 genes (*atpB*, *atpE*, *atpF*, *atpI*, *cemA*, *clpP*, *matK*, *ndhC*, *ndhE*, *ndhF*, *ndhH*, *ndhK*, *petA*, *psaB*, *psbH*, *psbJ*, *psbN*, *rps14*, *rps16*) (Fig. 7 and Additional File 4: Supplementary Table S9).

### Phylogenetic analysis

**Fig. 8:**
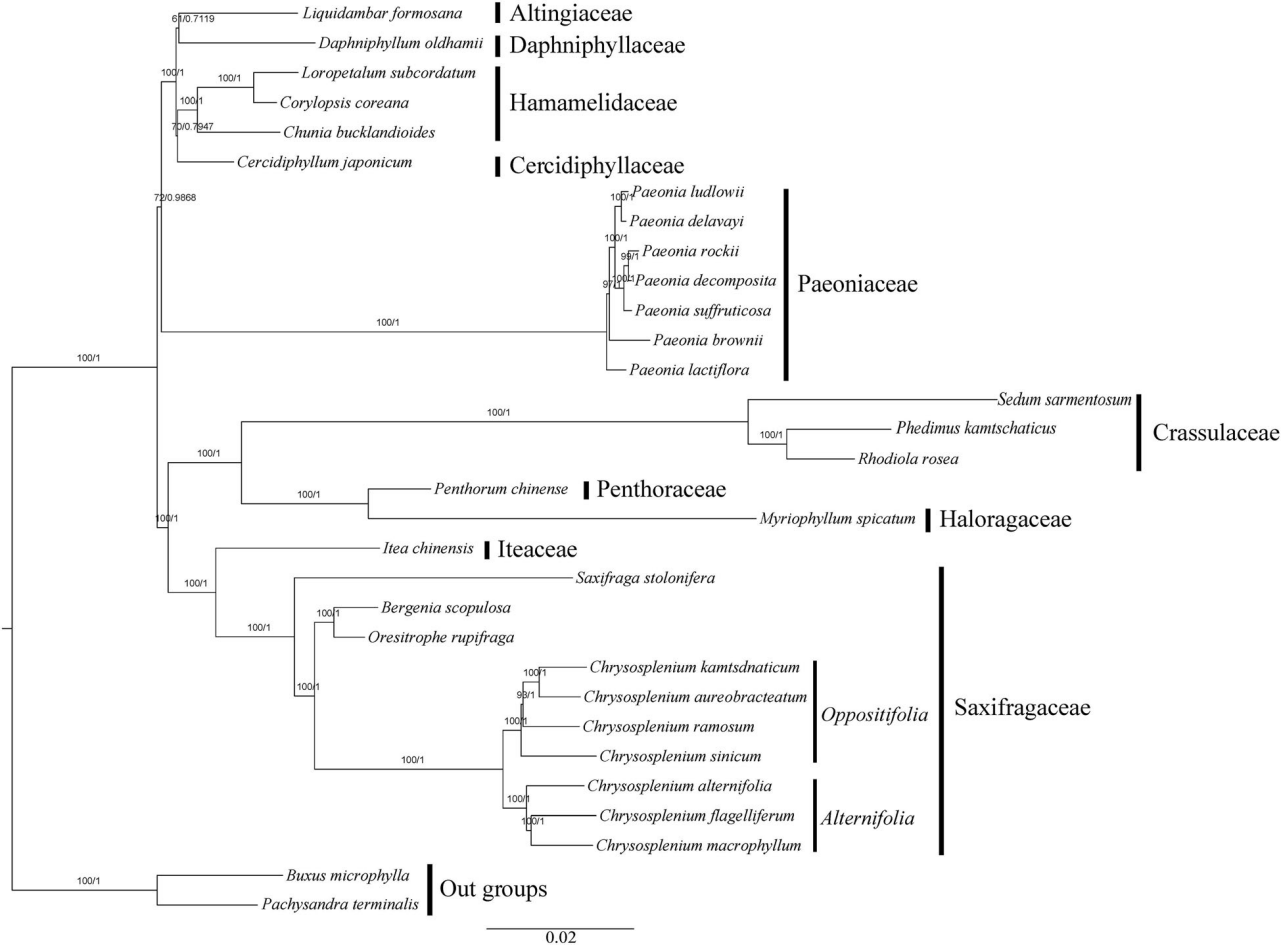
Phylogenetic tree reconstructed by Maximum likelihood (ML) and Bayesian inference (BI) analysis based on the whole chloroplast protein-coding genes of 31 species. The ML topology is indicated with ML bootstrap support values and BI posterior probabilities at each node

Phylogenetic analyses yielded a well-supported phylogeny of Saxifrageles with most of the nodes having maximum likelihood (ML) bootstrap support values > 95 and bayesian inference (BI) posterior probabilities =1 (Fig. 8). The topologies yielded from ML analysis and BI analysis were completely identical. The topology of Saxifragales in our study was similar to the APG IV system [6] with Saxifragaceae closer to Iteaceae, phylogenetically. And, *Chrysosplenium* was divided into two clades corresponding to the two subgenera (*Alternifolia* and *Oppositifolia*) in our phylogenetic tree.

## Discussion

Gene numbers were slightly different due to the loss of *rpl32* or transfer of the gene to the nucleus [22–26]. The gene transfer and its dormant expression in Alternifolia of *Chrysosplenium* could be explained by the decreased demand on photosynthesis and plastid translational capacity, which increased the success rate of gene transfer to the nucleus [27]. Based on the previous study and our results, we speculate the *rpl32* gene might transfer to the nucleus due to the adaptation for the specific habitat of *Chrysosplenium*, although this hypothesis remains to be verified by experiments.

Lower GC contents in *Chrysosplenium* chloroplast genomes compared to other Saxifragaceae members can be explained by the natural selection [28]. DNA sequences of closely related species from different environments show marked differences in GC content, which has a direct impact on the amino acid sequences of the proteins in the respective environments [29]. Genes with low GC contents are more prone to be transcribed than those with high GC content as GC pairs have three hydrogen bonds, making them more stable than AT pairs with two hydrogen bonds [30]. Therefore, the selective pressure of the unique habitat of *Chrysosplenium* species (insufficient light energy) resulted in the lower overall GC contents and GC3 contents in their chloroplast genomes.

Compared with other genera within Saxifragaceae, the genus has the lowest light requirement. Among the 79 chloroplast genes in plants, 46 are related to photosynthesis pathway (Table 2) [31]. Genes related to a specific environment are normally assumed to be under positive selection [32]. This assumption was widely used to detect genes related to environmental adaptation [33]. Our expectation was that the 46 genes were under positive selection. However, the lower Ka/Ks ratios at the chloroplast genome level within the *Chrysosplenium* species compared to non-*Chrysosplenium* species indicated that most genes were subjected to purifying selection to retain conserved functions in the *Chrysosplenium*. In the opposite environment, sunlight, including UV radiation, induces DNA damage, mutations and rearrangements [33, 34], which may contribute to an increase in mutation rates. Moreover, it has been proposed that more solar radiation and higher temperatures increase metabolism and growth rates, shortening generation times and increasing mutation rates [35]. Meanwhile, low light is also a stress to the plants which can increase the mutation rates. As one of the most prevalent form in natural selection, purifying selection constantly sweeps away deleterious mutations in population. Therefore, the purifying selection on most chloroplast genes within *Chrysosplenium* would be evolutionary result of the preservation of the adaptive characteristics of *Chrysosplenium* species.

Plants have a variety of strategies to adapt to the environment; therefore, multiple genes may have been subjected to positive selection in *Chrysosplenium* during its adaptation. The gene *matK* is transcribed from the sole intact plastid group IIA intron ORF localized between the exons coding for the lysine-tRNA (*trnK-UUU*). In contrast to other group IIA ORFs, *matK* has lost domains assigned to a reverse transcriptase and endonuclease function [31, 36]. *matK* is usually used as a phylogenetic signal that can resolve evolutionary relationships because of its the high nucleotide and amino acid substitution rates [37, 38]. However, an extremely significant positive selection site at the 117S loci in *matK* of *Chrysosplenium* suggests this positive selection fixes beneficial variations within *Chrysosplenium.* The gene *ycf2* is the largest chloroplast gene reported in angiosperms [39]. *ycf2* has become a useful gene for assessing sequence variation and evolution in plants [40]. Positive selection of *ycf2* was also found to be involved in adaptation in other species [41]. However, due to the extremely high Ka/Ks value (ѡ = 231.51421) and unknown function, *ycf2* is a valuable resource for future research of the adaptive evolution of *Chrysosplenium*. Further functional studies on the adaptive amino acid sites of chloroplast genes in *Chrysosplenium* are needed.

## Conclusions

In this study, we sequenced the chloroplast genomes of six *Chrysosplenium* species and revealed the chloroplast genomic features between the *Oppositifolia* (*C. macrophyllum*, *C. flagelliferum*, *C. alternifolium*, and *C. ramosum*) and *Alternifolia* (*C. ramosum*, *C. kamtschaticum*, and *C. sinicum*) subgenera. In addition, we combined these six sequences with the previously reported chloroplast genomes of *C. aureobracteatum* (*Oppositifolia*), *S. stolonifera* (Saxifragaceae), *B. scopulosa* (Saxifragaceae), and *O. rupifraga* (Saxifragaceae). We discussed the comprehensive features of the chloroplast genomes, such as gene content and GC content, in these seven species of *Chrysosplenium*. All the species of Saxifragaceae shared similar genome structures, whereas the seven species of *Chrysosplenium* showed a lower average GC content, indicating selective pressure in their unique habitats. At the chloroplast genome level, the Ka/Ks ratios of the individual sequences showed that *Chrysosplenium* species were subjected to purifying selection compared to the non-*Chrysosplenium* species. At the level of amino acid sites, we found that *matK* and *ycf2* were under positive selection with both high posterior probability and statistical significance. Other 15 genes with lower posterior probability involved in photosynthesis out of 19 were also possibly subjected to positive selection via a change of amino acid sites, which may be the adaptive response to its moist and shaded habitat. Using the protein-coding sequences from the whole chloroplast genome of 31 species, the robust consensus of phylogenetic trees reconstructed with both ML and BI algorithms suggested that *Chrysosplenium* species are sister to *B. scopulosa* and *O. rupifraga* within Saxifragaceae of Saxifragales. Also, our results supported the classification of the genus into two subgenera based on the morphology of opposite leaves or alternate leaves. These findings will be valuable for further study of the chloroplast genomes of *Chrysosplenium* species and provide valuable resources for studies of plant adaptation to low light conditions.

## Methods

### Sampling and sequencing

To represent the *Chrysosplenium*, six species were selected based on their morphological characteristics: *C. macrophyllum*, *C. flagelliferum*, and *C. alternifolium* belonging to the *Alternifolia* subgenus, and *C. ramosum*, *C. kamtschaticum*, and *C. sinicum* belonging to the *Oppositifolia* subgenus. Among the six species from wild, three were collected from China and three were from Japan (See details in Additional File 2: Supplementary Table S1). Due to a high content of secondary metabolites, the chloroplast DNA of *C. macrophyllum* was extracted using a high-salt method [42]. To get a complete chloroplast genome, which can be used as a reference in assembling chloroplast genome of the other five species, *C. macrophyllum* was sequenced using the PacBio Sequel I platform at Frasergen (Wuhan, China) and the Illumina Hiseq 2500 at the Novogene Company (Beijing, China). The total genomic DNA for the other five *Chrysosplenium* species was extracted using a modified cetyltrimethylammonium bromide (CTAB) method [43] and sequenced using the Illumina Hiseq 2500 platform at the Novogene Company (Beijing, China).

### Chloroplast genome assembly and annotation

The sequencing of the chloroplast DNA of *C. macrophyllum* with the PacBio Sequel I platform generated 218,330 reads with the N50 of 4452 bp. De novo genome assembly was conducted using Canu (v1.5) [44], which produced 4028 contigs with an N50 of 5011 bp. To discard nuclear DNA sequences, we aligned the contigs to a whole-chloroplast reference genome with the Burrow-Wheeler Aligner bwa [45]. Then the contigs were polished with Arrow implemented in SMRT Link v6.0.0. Finally, the draft chloroplast genome was manually adjusted based on the two inverted repeats and scaffolds assembled from the Illumina Hiseq 2500 platform.

Sequence data for the other five species generated from the Illumina Hiseq 2500 platform were processed to remove the low-quality reads and adaptors. The clean reads were aligned to the complete chloroplast genome of *C. macrophyllum* with bwa-0.7.12 [45]. The aligned reads were then selected for de novo assembly with ABYSS-2.0.2 [46] after the optimal K-mer was chosen with the software kmergenie [47]. Then, the contigs were connected with Sequencher 5.4.6 and scaffolded again with the original data by the software SSPACE_Standard_v3.0 [48]. Last, the assembled scaffolds were manually adjusted based on the two inverted repeats and verified by Sanger sequencing (Additional File 2: Supplementary Table S2). We also assembled these scaffolds with GetOrganelle [49] to validate the ones assembled with ABYSS-2.0.2.

Gene annotation was performed using CPGAVAS2 [50] and PGA [51]. The different annotations of protein-coding sequences were confirmed using BLASTx. The tRNAs were checked with tRNAscan-SE v2.0.3 [52]. Final chloroplast genome maps were drawn using OGDRAW [53].

To identify the nuclear homologs of chloroplast *rpl32* (*Cp_rpl32*) in *C. sinicum*, the protein sequence of *Cp_rpl32* was subjected to a BLASTp analysis against our the whole protein sequences from our nuclear genome of *C. sinicum* with the threshold of 30% identity, and the nuclear *rpl32* genes were further annotated via the BLASTp of these potential nuclear homologs against NCBI non-redundant protein database with the threshold of 30% identity and an e-value <1E− 10.

### Analysis of GC content, nucleotide diversity, and repeat content

We accessed chloroplast genome sequences of *Chrysosplenium aureobracteatum* (MG878089; Saxifragaceae; *Chrysosplenium*), *Saxifraga stolonifera* (MH191389; Saxifragaceae; *Saxifraga*), *Bergenia scopulosa* (KY412195; Saxifragaceae; *Bergenia*), and *Oresitrophe rupifraga* (MF774190; Saxifragaceae; *Oresitrophe*) from GenBank to compare the features among these chloroplast genomes in Saxifragaceae. The nucleotide diversity (Pi) among the seven species of *Chrysosplenium* was calculated using the software DnaSP v6.12.03 [54]. The GC content of the whole chloroplast genome and the third position GC content of codons for all ten species were calculated using an in-house Python script. The simple sequence repeats (SSRs) were detected using the MIcroSAtellite (MISA) identification tool with the minimum repeat number set at 10, 5, 4, 3, 3, and 3 for mono-, di-, tri-, tetra-, penta-, and hexanucleotides, respectively. We also identified tandem repeat sequences using REPuter [55] with minimal repeats of more than 30 bp and hamming distances of less than 3 bp.

### Boundary regions and comparative analysis

The contraction or expansion between boundary regions of the chloroplast genome in each species was drawn by IRscope [56]. To compare the conservation of each gene, we visualized the results with mVISTA through two alignment programs: LAGAN, which produces true multiple alignments regardless of whether they contain inversions or not, and Shuffle-LAGAN, which can detect rearrangements and inversions in sequences [57].

### Selective pressure estimation

We carried out selective pressure estimation for the 6 species of *Chrysosplenium* and 25 species of non-*Chrysosplenium* with two strategies: calculation based on pairwise comparison and calculation based on the branch-site model:

All protein-coding sequences (CDSs) from each of the 31 species were concatenated into a super matrix for inferring phylogenetic tree. Then, species vs. species Ka/Ks ratio was estimated. In addition, the Ka/Ks ratio was estimated for each of the 79 genes within *Chrysosplenium* separately. The CDS for each gene was translated to amino acid sequences, which were aligned with MEGA7 [58]. Then, the corresponding CDS were aligned according to the amino acid sequences. Lastly, Ka/Ks ratios were calculated using the KaKs-calculator v 2.0 [59]. Genes with 1 < Ka/Ks ratio < 45 were considered as under positive selection; genes with Ka/Ks ratio < 1 were considered as under purifying selection. The ratio > =45 or NA indicates that the gene has few nonsynonymous sites/substitutions, and was not considered in our analysis.

A total of 66 CDSs presented in all the analysed species, and were used for identification of positive selection using the branch-site model [32]. CDSs of each gene were aligned according to their amino acid sequences with MEGA7 [58]. The branch-site model in the program codeml of the PAML v4.9 package [60] was used to assess potential positive selection in *Chrysosplenium* that was set as the foreground branch. Selective pressure is measured by the ratio (ω) of the nonsynonymous substitution rate (dN) to the synonymous substitutions rate (dS). A neutral branch-site model (Model = 2, NSsites = 2, Fix = 1, and Fix ω = 1) and an alternative branch-site model (Model = 2, NSsites = 2, and Fix = 0) were applied separately. The right-tailed Chi-square test was used to compute *p*-values based on the difference of log-likelihood values between the two models with one degree of freedom. Moreover, BEB method [61] was implemented to calculate the posterior probabilities for amino acid sites that are potentially under positive selection. A gene with a *p*-value < 0.05 and ω > 1 was considered as a positively selected gene. An amino acid site with posterior probabilities > 0.95 was considered as positively selected.

### Phylogenetic analyses

To construct a phylogeny of *Chrysosplenium*, 29 species of Saxifrageles and two Buxaceae species (as outgroups) were selected (see Additional file 2: Supplementary Table S3 for details). The whole-chloroplast protein-coding genes of these 31 species were aligned with MUSCLE v3.8.31 [62]. The best-fitting nucleotide substitution model was determined using the Akaike Information Criterion in the model-finder IQ-TREE [63]. ML analysis was performed using IQ-TREE with the best model of GTR + F + R4 and 1000 bootstrap replicates, and BI analysis was performed in MrBayes 3.2.6 [64] using the Markov Chain Monte Carlo method with 200,000 generations and sampling trees every 100 generations. The first 20% of trees were discarded as burn-in with the remaining trees being used for generating a consensus tree.

## Supplementary information

**Additional File 1 **The protein sequences (A) and expression values in leaf (B) of both chloroplast *rpl32* (*Cp_rpl32*) and its nuclear homolog (*Nu_rpl32*) in *C. sinicum.*

**Additional File 2.** Supplementary Tables S1-S6.

**Additional File 3 **Comparison of nucleotide diversity (Pi) between the chloroplast genomes of *Chrysosplenium*.

**Additional File 4.** Supplementary Tables S7-S11.

**Additional File 5.** Analyses of repeat sequences in the ten species of Saxifragaceae. (A) The analysis of simple sequence repeats (SSRs) in chloroplast genomes of Saxifragaceae. (B) The repeat types in Saxifragaceae.

**Additional File 6.** The comparative analysis with Shuffle-LAGAN program of the whole chloroplast genome of seven different species from the family of Saxifragaceae.

## Data Availability

All chloroplast genomes used in this study can be found in Genbank and their Genbank accessions can be found in Additional File 2: Supplementary Table S1 and Additional File 2: Supplementary Table S3. The whole protein sequences of the nuclear genome of *C. sinicum* in our study are archived at the Dryad Digital Repository (10.5061/dryad.jdfn2z38m). The other data sets generated in this study are included within the article and additional files. All materials used or generated during the study are kept in our laboratory. Most figures were completed by the open source software, R package; some figures were done by the relevant software with the proper citations.

## Abbreviations

LSC: Large single copy region
IR: Inverted repeat region
SSC: Small single copy region
PCR: Polymerase chain reaction
tRNAs: Transport RNAs
rRNAs: Ribosomal RNAs
Ka/Ks: The rate of non-synonymous substitutions to the rate of synonymous substitutions
SSRs: Simple sequence repeats
ML: Maximum likelihood
BI: Bayesian inference
BEB: Bayes empirical bayes
IGS: Intergenic spacer region
FPKMs: Fragments per kilobase of exon per million reads
