# Supplementary figures and images for: Analysis of six chloroplast genomes provides insight into the evolution of Chrysosplenium (Saxifragaceae)
Source: BMC Genomics. 2020 Sep 10;21:621. doi: 10.1186/s12864-020-07045-4 (PMC7488271; doi:10.1186/s12864-020-07045-4)

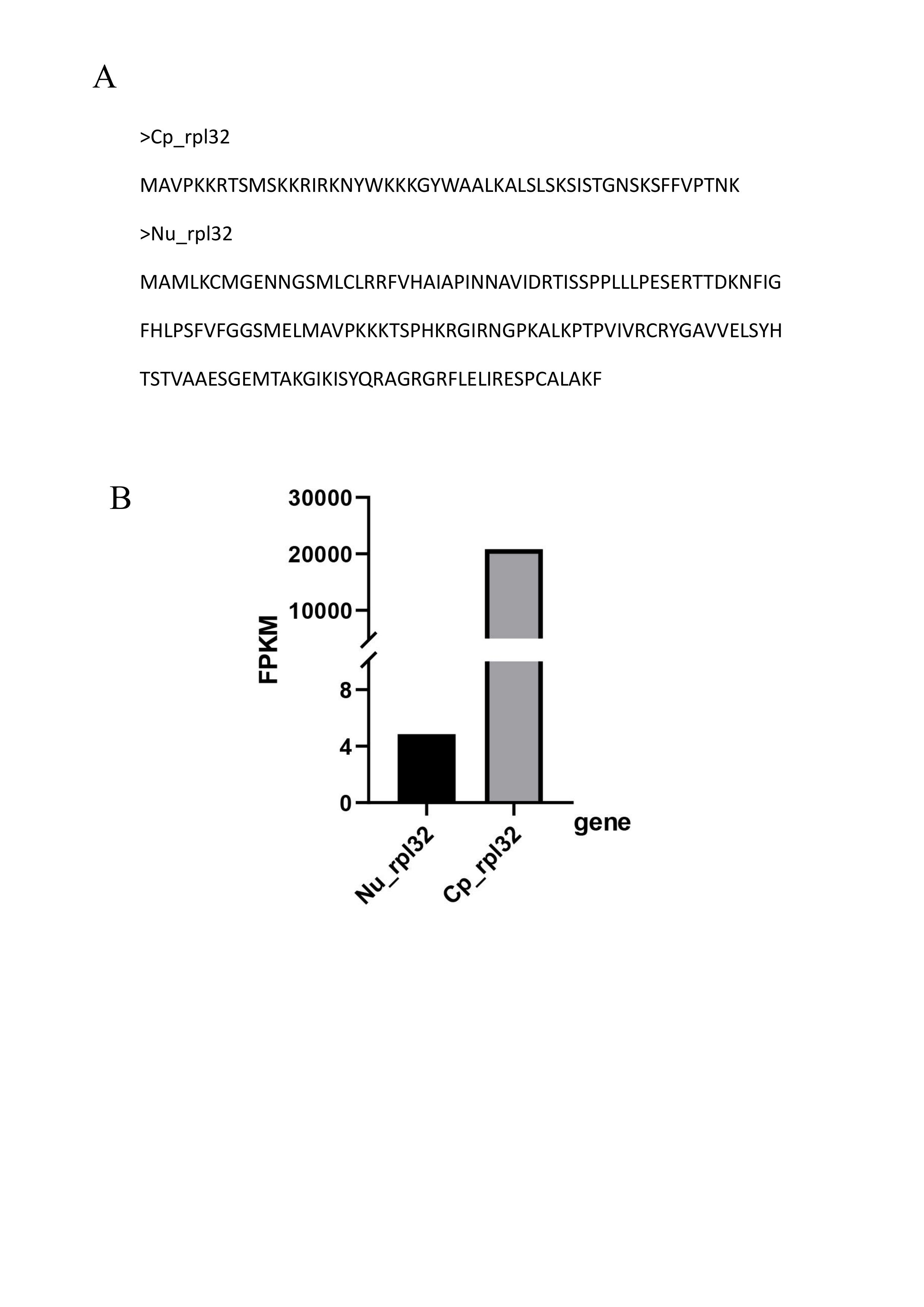

Supplement: Supplementary file 1 — Additional File 1 The protein sequences (A) and expression values in leaf (B) of both chloroplast rpl32 (Cp_rpl32) and its nuclear homolog (Nu_rpl32) in C. sinicum. [file 12864_2020_7045_MOESM1_ESM.jpg]

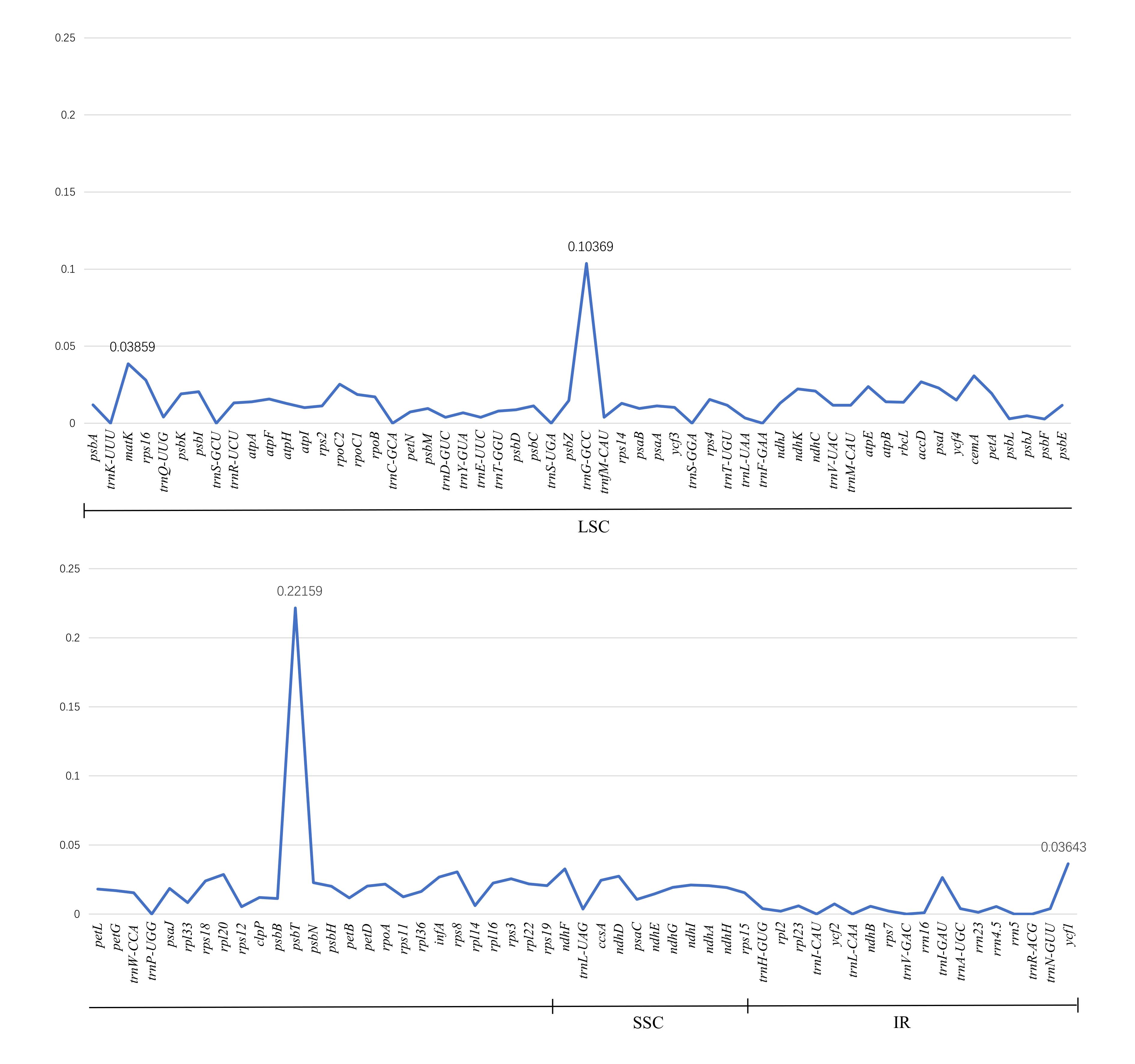

Supplement: Supplementary file 3 — Additional File 3 Comparison of nucleotide diversity (Pi) between the chloroplast genomes of Chrysosplenium. [file 12864_2020_7045_MOESM3_ESM.jpg]

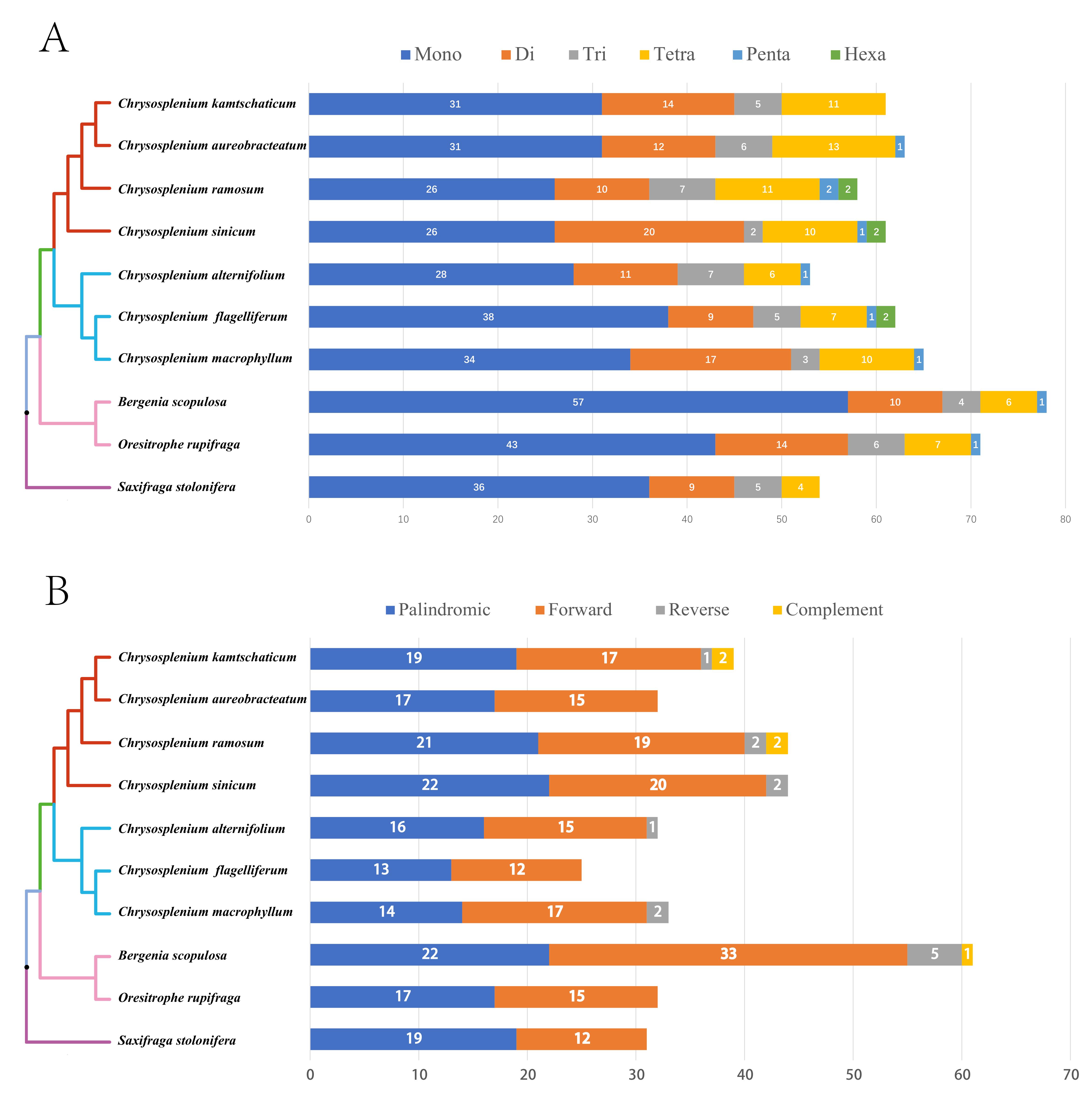

Supplement: Supplementary file 5 — Additional File 5. Analyses of repeat sequences in the ten species of Saxifragaceae. (A) The analysis of simple sequence repeats (SSRs) in chloroplast genomes of Saxifragaceae. (B) The repeat types in Saxifragaceae. [file 12864_2020_7045_MOESM5_ESM.jpg]

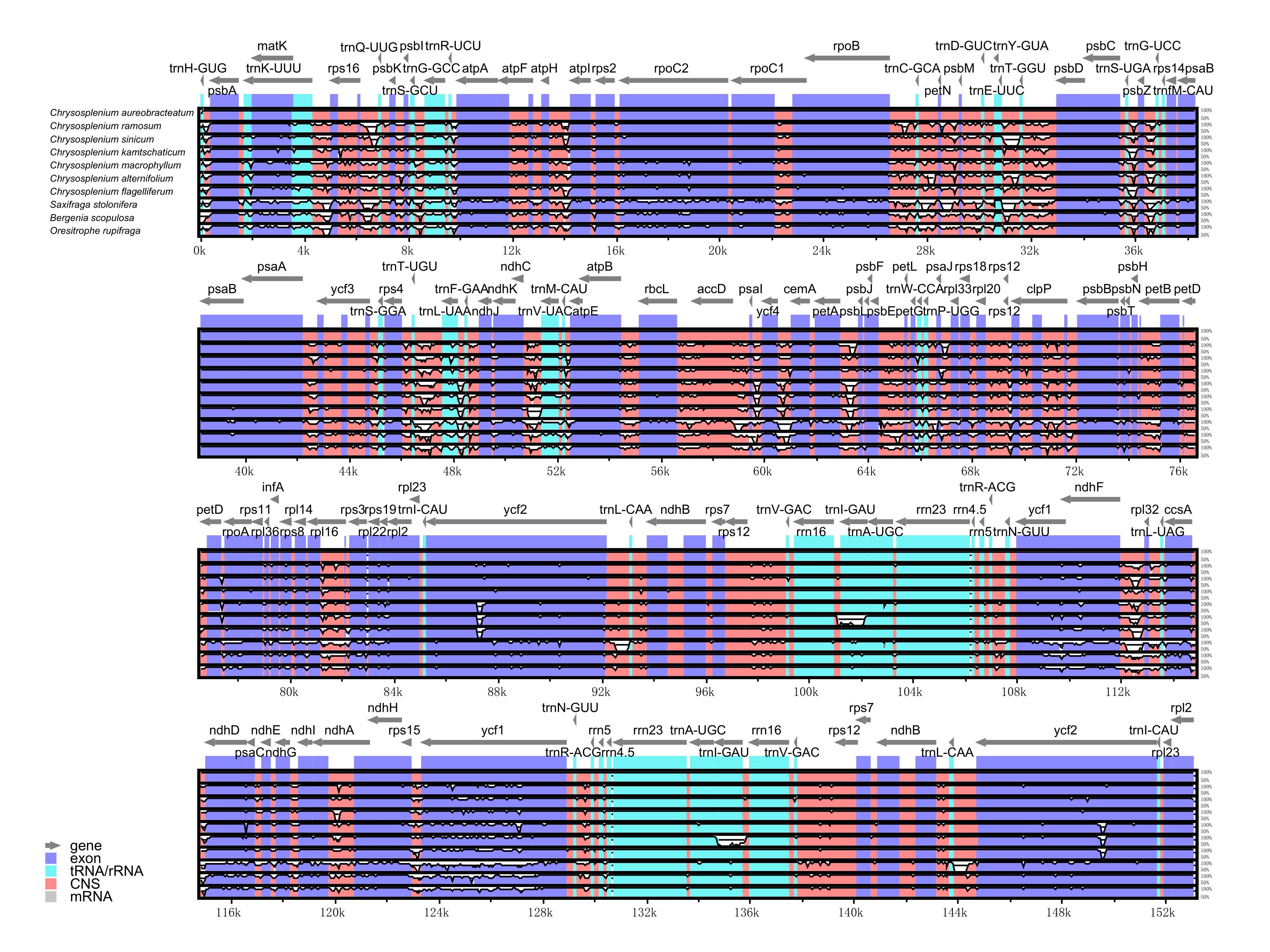

Supplement: Supplementary file 6 — Additional File 6. The comparative analysis with Shuffle-LAGAN program of the whole chloroplast genome of seven different species from the family of Saxifragaceae. [file 12864_2020_7045_MOESM6_ESM.jpg]
