# Supplementary material for: Analysis of six chloroplast genomes provides insight into the evolution of Chrysosplenium (Saxifragaceae)
Source: BMC Genomics. 2020 Sep 10;21:621. doi: 10.1186/s12864-020-07045-4 (PMC7488271; doi:10.1186/s12864-020-07045-4)
Supplement: Supplementary file 2 — Additional File 2. Supplementary Tables S1-S6. [file 12864_2020_7045_MOESM2_ESM.docx]

**Supplementary Table S1** Information for *Chrysosplenium* sample collections in this study

| Species | Subgenera | GenBank number | Location | Voucher specimens* | Longitude | Latitude | Altitude |
| --- | --- | --- | --- | --- | --- | --- | --- |
| *Chrysosplenium macrophyllum* | *Alternifolia* | MK973001 | Hubei, China | BD2017030507344 | 110° 15' 26" | 30° 44' 04" | 1426 |
| *Chrysosplenium flagelliforum* | *Alternifolia* | MN729584 | Tochigi-ken, Japan | RG2019032810002 | 139° 36' 01'' | 36° 45' 32'' | 640 |
| *Chrysosplenium alternifolium* | *Alternifolia* | MT362050 | Shimane-ken, Japan | DG2019032310003 | 132° 36' 11'' | 35° 08' 22'' | 448 |
| *Chrysosplenium kamtsdnaticum* | *Oppositifolia* | MT371065 | Shimane-ken, Japan | DG2019032310004 | 132° 37' 26'' | 35° 08' 46'' | 727 |
| *Chrysosplenium ramosum* | *Oppositifolia* | MK973002 | Jilin, China | SJH2017052107372 | 127° 30' 30" | 42° 10' 27" | 400 |
| *Chrysosplenium sinicum* | *Oppositifolia* | MT362051 | Hunan, China | TPS2017042407504 | 110° 04' 20" | 29° 42' 21" | 2011 |

*The voucher specimens were deposited at the Herbarium of South-Central University for Nationalities.

**Supplementary Table S2** Primers used for gap closure in this study

| Species | Primer name | Primer sequence |
| --- | --- | --- |
| *Chrysosplenium macrophyllum* | 1F | AATAAATGAGTTCGTCTT |
|  | 1R | CATAAATCGTTGGTGGTT |
|  | 2F | TCTTTCTGGTAGGTATCC |
|  | 2R | AATAATGCCTATGACTAA |
|  | 3F | TTTTGGAATTGATACATTGG |
|  | 3R | AATGCCCCAAAAAGCCATTT |
|  | 4F | TGGGGTGAACCAGAAAAG |
|  | 4R | GGGCAAGAGGAAGAAACT |
| *Chrysosplenium flagelliforum* | 1F | GTTCGGAACCTTTCTTTG |
|  | 1R | CTCACGTCAGTGGCATAT |
|  | 2F | GCTCGGTTTCCATTTCGG |
|  | 2R | CTATTCAGCGGGCGTGGT |
|  | 3F | TGTTGACCTAAAGCGTAT |
|  | 3R | AAGCGTCCTGTAGTAAGA |
|  | 4F | AGTATCTGGGGTAGTAGT |
|  | 4R | TTATAAGTCGAATAGAGG |
| *Chrysosplenium alternifolium* | 1F | GAACCTCCCACTAATGAC |
|  | 1R | TATGAACCCTGTAGACCA |
|  | 2F | AGCGACTTCGTCTGTTGG |
|  | 2R | ACTAGATAATCACTGGGTT |
| *Chrysosplenium kamtsdnaticum* | 1F | TAACCTCCCACTAATGAA |
|  | 1R | CAGCAACAGTCGGACAAG |
|  | 2F | GGGGATTGCCCATTCTGT |
|  | 2R | TTTCTCGAACGGGCCTTA |
| *Chrysosplenium ramosum* | 1F | GGGAGAAGGATGGATTGT |
|  | 1R | AAGAGGGGTCATGGAAAG |
| *Chrysosplenium sinicum* | 1F | CATACGATGTCTGTCTCCCACC |
|  | 1R | CCAATAATTCGCTAATCCAAGA |
|  | 2F | GGCTGCTTTAGCTGTCTT |
|  | 2R | GCATCGGATCTACTCTTGT |

**Supplementary** **Table S3** Summary for the chloroplast genomes of 25 species

| Species | GenBank number | Journal | Size | Lacking gene |
| --- | --- | --- | --- | --- |
| *Bergenia scopulosa* | NC_036061.1/KY412195.1 | Conserv Genet Resour (2017) | 156,041 |  |
| *Oresitrophe rupifraga* | NC_037514.1/MF774190.1 | BMC Genomics (2018) | 156,738 |  |
| *Saxifraga stolonifera* | NC_037882.1/MH191389.1 | Mol. Phylogenet. Evol. (2018) | 151,066 | *psbB* |
| *Liquidambar formosana* | NC_023092.1/KC588388.1 | Genome Biol Evol (2013) | 160,410 |  |
| *Cercidiphyllum japonicum* | NC_037940.1/MG605672.1 | Conserv Genet Resour (2018) | 159,854 | *psbM* |
| *Phedimus kamtschaticus* | NC_037946.1/MG680403.1 | Mitochondrial DNA B Resour (2018) | 151,652 | *psaI, psbI, rpl16* |
| *Rhodiola rosea* | NC_041671.1/MH410216.1 | Mitochondrial DNA B Resour (2018) | 151,348 | *psbZ* |
| *Sedum sarmentosum* | NC_023085.1/JX427551.1 | Genome Biol Evol (2013) | 150,448 | *psbZ* |
| *Daphniphyllum oldhamii* | NC_037883.1/MH191390.1 | Mol. Phylogenet. Evol. (2018) | 160,137 |  |
| *Myriophyllum spicatum* | NC_037885.1/MH191392.1 | Mol. Phylogenet. Evol. (2018) | 158,860 | *psbZ, rpl22* |
| *Chunia bucklandioides* | NC_041163.1/MG644608.1 | Conserv Genet Resour (2019) | 159,814 | *infA, rpl22* |
| *Corylopsis coreana* | NC_040141.1/MG835449.1 | Conserv Genet Resour (2018) | 159,398 |  |
| *Loropetalum subcordatum* | NC_037694.1/MG457805.1 | Conserv Genet Resour (2018) | 158,706 |  |
| *Itea chinensis* | NC_037884.1/MH191391.1 | Mol. Phylogenet. Evol. (2018) | 160,258 |  |
| *Paeonia brownii* | NC_037880.1/MH191385.1 | Mol. Phylogenet. Evol. (2018) | 152,228 | *infA, rpl22, rpl32, rps18* |
| *Paeonia decomposita* | NC_039425.1/MG571273.1 | Conserv Genet Resour (2018) | 152,601 | *infA, psbL, rpl22, rpl32* |
| *Paeonia delavayi* | NC_035718.1/KY817591.1 | Conserv Genet Resour (2017) | 154,405 | *infA, psbL, rpl22, rpl32* |
| *Paeonia lactiflora* | NC_040983.1/MG897127.1 | Mitochondrial DNA B Resour (2018) | 152,747 | *infA, rpl22, rpl32* |
| *Paeonia ludlowii* | NC_035623.1/KY817592.1 | Conserv Genet Resour (2017) | 152,687 | *infA, rpl22, rpl32* |
| *Paeonia rockii* | NC_037772.1/MF488719.1 | Conserv Genet Resour (2017) | 152,821 | *infA, rpl22, rpl32, rps18* |
| *Paeonia suffruticosa* | NC_037879.1/MH191384.1 | Mol. Phylogenet. Evol. (2018) | 153,119 | *infA, rpl22, rpl32* |
| *Penthorum chinense* | NC_023086.1/JX436155.1 | Genome Biol Evol (2013) | 156,686 | *psbB, psbZ* |
| *Buxus microphylla* | NC_009599.1/EF380351.1 | Mol. Phylogenet. Evol. (2007) | 159,010 |  |
| *Pachysandra terminalis* | NC_029433.1/KU204904.1 | Mol. Phylogenet. Evol. (2015) | 160,623 |  |
| *Chrysosplenium aureobracteatum* | MG878089 | Mitochondrial DNA B Resour (2018) | 153,102 |  |

**Supplementary Table S4** Statistics of the intron number of six *Chrysosplenium* chloroplast genomes

| Species | One intron | Two introns |
| --- | --- | --- |
| *Chrysosplenium macrophyllum* | *trnK-UUU, rps16, trnG-GCC, atpF, rpoC1, trnL-UAA, trnV-UAC, petB, petD, rpl16, ndhB, trnI-GAU, trnA-UGC, ndhA* | *rps12, ycf3, clpP* |
| *Chrysosplenium flagelliforum* | *trnK-UUU, rps16, trnG-GCC, atpF, rpoC1, trnL-UAA, trnV-UAC, petB, petD, rpl16, ndhB, trnI-GAU, trnA-UGC, ndhA* | *rps12, ycf3, clpP* |
| *Chrysosplenium alternifolium* | *trnK-UUU, rps16, trnG-GCC, atpF, rpoC1, trnL-UAA, trnV-UAC, petB, petD, rpl16, ndhB, trnI-GAU, trnA-UGC, ndhA* | *rps12, ycf3, clpP* |
| *Chrysosplenium kamtsdnaticum* | *trnK-UUU, rps16, trnG-GCC, atpF, rpoC1, trnL-UAA, trnV-UAC, petB, petD, rpl16, ndhB, trnI-GAU, trnA-UGC, ndhA* | *rps12, ycf3, clpP* |
| *Chrysosplenium ramosum* | *trnK-UUU, rps16, trnG-GCC, atpF, rpoC1, trnL-UAA, trnV-UAC, petB, petD, rpl16, ndhB, trnI-GAU, trnA-UGC, ndhA* | *rps12, ycf3, clpP* |
| *Chrysosplenium sinicum* | *trnK-UUU, rps16, trnG-GCC, atpF, rpoC1, trnL-UAA, trnV-UAC, petB, petD, rpl16, ndhB, trnI-GAU, trnA-UGC, ndhA* | *rps12, ycf3, clpP* |

**Supplementary Table S5** Statistics of the whole GC contents, CDS GC contents and GC contents of the first (GC1), second (GC2) and third (GC3) position in codons

| **Species** | **Whole GC contents** | **CDS GC contents** | **GC1** | **GC2** | **GC3** |
| --- | --- | --- | --- | --- | --- |
| *Chrysosplenium macrophyllum* | 37.46% | 37.70% | 45.59% | 38.00% | 29.52% |
| *Chrysosplenium flagelliforum* | 37.43% | 37.65% | 45.39% | 37.93% | 29.64% |
| *Chrysosplenium alternifolium* | 37.47% | 37.80% | 45.79% | 38.13% | 29.49% |
| *Chrysosplenium kamtsdnaticum* | 37.41% | 37.64% | 45.64% | 37.97% | 29.32% |
| *Chrysosplenium ramosum* | 37.38% | 37.72% | 45.58% | 38.01% | 29.57% |
| *Chrysosplenium sinicum* | 37.26% | 37.58% | 45.38% | 38.01% | 29.35% |
| *Chrysosplenium aureobracteatum* | 37.35% | 37.65% | 45.50% | 38.04% | 29.41% |
| *Bergenia scopulosa* | 37.77% | 38.01% | 42.94% | 39.97% | 31.12% |
| *Oresitrophe rupifraga* | 37.75% | 38.10% | 45.94% | 38.35% | 30.02% |
| *Saxifraga stolonifera* | 37.83% | 37.84% | 45.52% | 38.00% | 29.99% |

**Supplementary Table S6** The statistics of nucleotide diversity (Pi) between the species of *Chrysosplenium*

| Region | Gene | Pi | Gene | Pi | Gene | Pi | Gene | Pi |
| --- | --- | --- | --- | --- | --- | --- | --- | --- |
| LSC | *psbA* | 0.01199 | *trnY-GUA* | 0.006803 | *trnV-UAC* | 0.01174 | *rpl20* | 0.02863 |
|  | *trnK-UUU* | 0 | *trnE-UUC* | 0.003914 | *trnM-CAU* | 0.01174 | *rps12* | 0.00538 |
|  | *matK* | 0.03859 | *trnT-GGU* | 0.007937 | *atpE* | 0.0238 | *clpP* | 0.01196 |
|  | *rps16* | 0.02797 | *psbD* | 0.00879 | *atpB* | 0.01394 | *psbB* | 0.01122 |
|  | *trnQ-UUG* | 0.00397 | *psbC* | 0.01126 | *rbcL* | 0.01359 | *psbT* | 0.22159 |
|  | *psbK* | 0.019 | *trnS-UGA* | 0 | *accD* | 0.02692 | *psbN* | 0.02273 |
|  | *psbI* | 0.02042 | *psbZ* | 0.01481 | *psaI* | 0.02282 | *psbH* | 0.02012 |
|  | *trnS-GCU* | 0 | *trnG-GCC* | 0.10369 | *ycf4* | 0.01502 | *petB* | 0.01163 |
|  | *trnR-UCU* | 0.01323 | *trnfM-CAU* | 0.00386 | *cemA* | 0.03082 | *petD* | 0.02024 |
|  | *atpA* | 0.01391 | *rps14* | 0.01298 | *petA* | 0.01931 | *rpoA* | 0.02168 |
|  | *atpF* | 0.01574 | *psaB* | 0.00958 | *psbL* | 0.00285 | *rps11* | 0.01247 |
|  | *atpH* | 0.01274 | *psaA* | 0.01119 | *psbJ* | 0.00488 | *rpl36* | 0.01637 |
|  | *atpI* | 0.01013 | *ycf3* | 0.01026 | *psbF* | 0.00278 | *infA* | 0.02678 |
|  | *rps2* | 0.01125 | *trnS-GGA* | 0 | *psbE* | 0.01164 | *rps8* | 0.03051 |
|  | *rpoC2* | 0.02528 | *rps4* | 0.0154 | *petL* | 0.01806 | *rpl14* | 0.00614 |
|  | *rpoC1* | 0.01871 | *trnT-UGU* | 0.01174 | *petG* | 0.01696 | *rpl16* | 0.02239 |
|  | *rpoB* | 0.01709 | *trnL-UAA* | 0.00340 | *trnW-CCA* | 0.01544 | *rps3* | 0.02548 |
|  | *trnC-GCA* | 0 | *trnF-GAA* | 0 | *trnP-UGG* | 0 | *rpl22* | 0.02179 |
|  | *petN* | 0.00741 | *ndhJ* | 0.013 | *psaJ* | 0.01852 | *rps19* | 0.02055 |
|  | *psbM* | 0.00952 | *ndhK* | 0.02222 | *rpl33* | 0.00829 |  |  |
|  | *trnD-GUC* | 0.00386 | *ndhC* | 0.02094 | *rps18* | 0.024 |  |  |
|  | **Pi average** | 0.01760 |  |  |  |  |  |  |
| SSC | *ndhF* | 0.03271 | *ndhD* | 0.0274 | *ndhG* | 0.01933 | *ndhH* | 0.01923 |
|  | *trnL-UAG* | 0.00357 | *psaC* | 0.01057 | *ndhI* | 0.02103 | *rps15* | 0.01548 |
|  | *ccsA* | 0.02452 | *ndhE* | 0.0146 | *ndhA* | 0.02051 |  |  |
|  | **Pi average** | 0.019 |  |  |  |  |  |  |
| IR | *trnH-GUG* | 0.00386 | *trnL-CAA* | 0 | *trnI-GAU* | 0.02646 | *trnR-ACG* | 0 |
|  | *rpl2* | 0.00202 | *ndhB* | 0.00561 | *trnA-UGC* | 0.00391 | *trnN-GUU* | 0.00397 |
|  | *rpl23* | 0.00591 | *rps7* | 0.00214 | *rrn23* | 0.00129 | *ycf1* | 0.03643 |
|  | *trnI-CAU* | 0 | *trnV-GAC* | 0 | *rrn4.5* | 0.00555 |  |  |
|  | *ycf2* | 0.00741 | *rrn16* | 0.00096 | *rrn5* | 0 |  |  |
|  | **Pi average** | 0.00586 |  |  |  |  |  |  |
